# Radial variations in xylem sap flux in a temperate red pine plantation forest

**DOI:** 10.1186/s13717-021-00295-4

**Published:** 2021-04-21

**Authors:** Alanna V. Bodo, M. Altaf Arain

**Affiliations:** 1 School of Earth, Environment and Society, McMaster University, Hamilton, ON Canada; 2 McMaster Centre for Climate Change, Hamilton, ON Canada

**Keywords:** Sap flow, Transpiration, Water use, Red pine, Temperate forest, Great Lakes region

## Abstract

**Background:**

Scaling sap flux measurements to whole-tree water use or stand-level transpiration is often done using measurements conducted at a single point in the sapwood of the tree and has the potential to cause significant errors. Previous studies have shown that much of this uncertainty is related to (i) measurement of sapwood area and (ii) variations in sap flow at different depths within the tree sapwood.

**Results:**

This study measured sap flux density at three depth intervals in the sapwood of 88-year-old red pine (*Pinus resinosa*) trees to more accurately estimate water-use at the tree- and stand-level in a plantation forest near Lake Erie in Southern Ontario, Canada. Results showed that most of the water transport (65%) occurred in the outermost sapwood, while only 26% and 9% of water was transported in the middle and innermost depths of sapwood, respectively.

**Conclusions:**

These results suggest that failing to consider radial variations in sap flux density within trees can lead to an overestimation of transpiration by as much as 81%, which may cause large uncertainties in water budgets at the ecosystem and catchment scale. This study will help to improve our understanding of water use dynamics and reduce uncertainties in sap flow measurements in the temperate pine forest ecosystems in the Great Lakes region and help in protecting these forests in the face of climate change.

## Background

Forests provide essential water-related ecosystem services through the regulation of the hydrologic cycle. Quantitative assessments of these ecosystem services have traditionally focused on direct water availability, failing to recognize the role of forests in moisture recycling from a supply, or “green water” perspective (Falkenmark and Rockstrom 2004; Ellison et al. 2017; Casagrande et al. 2021). At a global scale, 61% of atmospheric moisture is derived from terrestrial environments (Schneider et al. 2017), more than half of which comes from plant-transpiration (Schlesinger and Jasechko 2014; Wei et al. 2017). A thorough understanding of these processes may help to better characterize the complex linkages between forest ecosystems and climate change. Furthermore, the accurate quantification of these green water processes is becoming increasingly important for the development of the terrestrial ecosystem and hydrologic models (Guswa et al. 2014) as well as climate models (Marotzke et al. 2017).

Forest ecosystems play a dominant role in the transfer of ground water to the atmosphere through transpiration (Bonan 2008). Precise measurement of transpiration in forest ecosystems is essential to improve our understanding of their water-use and hence regional water resources. It will help to determine how forests may respond to future climatic changes, where future climate scenarios suggest increased air temperatures, more frequent and severe droughts, and longer growing seasons (Zhang et al. 2019), which may have a major impact on the transpiration and water-use in forest ecosystems.

Over the past 40 years scientists have developed several methods for estimating transpiration such as the water balance method, soil water budget, isotopes, and sap flow (Kool et al. 2014). The latter describes a technique of estimating the water flux through the conductive tissue of a plant (Vandegehuchte and Steppe 2013). There are several different methods to measure sap flow including the heat-pulse (Swanson and Whitfield 1981; Cohen et al. 1981; Green and Clothier 1988) and thermal-dissipation (Granier 1987) techniques. Both methods measure the difference in temperature between a heated and non-heated probe and create a dimensionless flow index (*K*). This flow index is then used to calculate sap flux density (*J*_*s*_; g H_2_O m^−2^ sapwood s^−1^), the flow rate per conductive area (Vandegehuchte and Steppe 2013). To estimate whole-tree water use from *J*_*s*_, the mass flow of sap in the conductive xylem sapwood (*F*) is calculated as the product of *J*_*s*_ and the sapwood area (*A*_*s*_) of the stem cross-section (*F = J*_*s*_ *A*_*s*_).

When scaling point-measurements to the whole-tree or stand level, many studies have identified within-tree variations in sap flow as one of the largest sources of error (Hatton et al. 1995; Oren et al. 1998; Clearwater et al. 1999; Wullschleger and King 2000; Zhang et al. 2015). To accurately understand whole-tree water use in the forest stand, scientists rely not only on the accuracy of these point-measurements but also on the methods used to scale-up the results to the tree- and stand-level (Clearwater et al. 1999). This includes an accurate estimation of sapwood depth and an understanding that *J*_*s*_ varies radially throughout the sapwood. Failure to take into consideration these radial differences has been shown to lead to errors as large as 300% when reporting whole-tree transpiration in the literature (Nadezhdina et al. 2002; Ford et al. 2004b).

In addition to variability throughout a cross-section of a tree, the sap flow method is subject to errors associated with uncertainties in the sapwood area. This is often due to the destructive nature of methods such as coring to determine sapwood depth. These errors can lead to insufficient contact area of the sampling probes in the sapwood and inaccuracies when scaling up point-measurements to the tree-level (Vandegehuchte and Steppe 2013; Lu et al. 2004). Few studies using sap flow methods to estimate tree- and stand-level transpiration characterize a radial profile within the sapwood (Ford et al. 2004b). Berdanier et al. (2016) examined studies published between 2013 and 2016 in which authors scaled up sap flow measurements and found 58% of studies assumed uniform flow throughout the sapwood, resulting in a large margin of error.

This study addresses many of the aforementioned uncertainties in sap flow measurements with the aim of decreasing error in the scaling-up process and further highlighting the importance of characterizing a radial profile of water conductance within the sapwood. The specific objectives of this study are (i) to measure the spatial (radial) variability in sap flow within the xylem sapwood of the red pine trees; (ii) to determine if a species-specific relationship exists between tree diameter and sapwood area; and (iii) to quantify errors associated with up-scaling of single point sap flow measurements to the whole tree level. We hypothesize that sap flux will be greatest closer to the cambium and decrease substantially toward the heartwood, where it is considered to be zero. The study results will help to develop a better understanding of the hydraulic conductivity of sapwood and help in up-scaling sap flow to the tree- and ecosystem levels.

Furthermore, this study is one of the first to study spatial variation in sap flow in red pine (*Pinus resinosa*)*—*an important plantation species in the region of southern Ontario. It is estimated that 70% of plantation forests in southern Ontario are comprised of red pine, a species which delivers valuable economic revenue from harvested timber (Kim 2020). These red pine plantations produce lumber which is extensively used for pulp wood and utility poles, and are also considered a principal solution for the restoration of wastelands into forests (LRC 2005).

## Methods

### Experimental site description

This study was conducted in a temperate red pine (*Pinus resinosa*) plantation forest located in the St. Williams Conservation Reserve (SWCR) (42°42′N, 80°21′W), 3.0 km north of Lake Erie, in southern Ontario, Canada. This 14-ha plantation stand is part of the Turkey Point Observatory or Turkey Point Flux Station (TPFS) and is referred to as TP31, where 31 represents 1931 when the stand was planted. Soils in this region are sandy and well-drained, with a low to moderate water holding capacity (McLaren et al. 2008; Beamesderfer et al. 2020). The TPFS consists of three different-aged coniferous plantations referred to in the literature as TP39, TP72, and TP02, one mixed deciduous site, TPD, one red pine plantation stand, TP31 (this study site), and an agricultural site (TPAg), where eddy covariance flux measurements have been made. These sites are also associated with Global Water Futures Program and Global Fluxnet. Further details of TPFS are given in Restrepo and Arain (2005), Peichl et al. (2010), Skubel et al. (2015), and Beamesderfer et al. (2020).

TP31 was established by planting red pine seedlings in furrowed rows, 2 m apart. In early 2014 the plantation was subject to a variable retention harvesting (VRH) regime to convert or restore this conifer plantation to a native mixed forest. VRH treatment included the division of the stand into 1-hectare plots and the application of different harvesting patterns to the plots. This study was conducted in three trees in the non-harvested (control) plots of this plantation. These trees are part of much larger VRH study where sap flow is being measured in 80 trees in 10 one-hectare plots comprising five management regimes or treatments. Because TP31 is a monoculture plantation stand with very small difference in tree DBH and structure, measuring radial difference in sap flow in three trees was adequate for this study.

### Meteorological information

The climate in southern Ontario is temperate with warm, humid summers and very cold winters. The region receives on average 1036 mm of precipitation per year, of which approximately 13% falls as snow (Environment Canada, Norms at Delhi, ON).

Local micrometeorological conditions were measured from two flux towers located at the white pine (*Pinus strobus*) plantation sites (TP39, TP74) within a 3-km radius of TP31. These towers are instrumented with eddy-covariance systems and weather stations, where continuous year-round measurements of sensible heat, latent heat, CO_2_, air temperature, humidity, photosynthetically active radiation (PAR), soil temperature, and soil moisture have been conducted since 2003.

### Sap flow measurements

Sap flow sensors were installed in three sample trees selected from a control (unharvested) plot at TP31 (Table 1). The sensors were self-manufactured, Granier-style thermal-dissipation sensors following Matheny et al. (2014) and Pappas et al. (2018). Each sensor consisted of two hollow needles, 20 mm in length, each containing a fine-wire, type T thermocouple at the midpoint (10 mm) of each needle. One of the needles was wrapped with insulated, constantan wire, which provided constant heating when connected to the self-made circuit board and supplied 12 V power. The needles were coated with thermal grease and inserted into a hollow, metal tube on the north side of the tree at breast height (1.3 m above the ground). The heated probe was installed 10 cm vertically above the non-heated probe.

**Table 1:** Biometric characteristics of sampled trees

Sample no.	Diameter at breast height (cm)	Height (m)	Crown area (m^2^)	Sapwood depth (cm)	Heartwood depth (cm)	Sapwood area (cm^2^)
1	31.7	25.5	145	7.5	7.9	549
2	28.7	21.8	112	6.9	7.0	453
3	32.2	26.4	168	7.7	7.9	568
**Mean**	**30.9**	**24.6**	**142**	**7.3**	**7.6**	**523**

In each tree, one sensor was installed in the outermost 0–20 mm of sapwood (from the edge of the cambium to 20 mm depth); a second sensor was installed from 20 to 40 mm depth and the third sensor was installed at a depth of 40–60 mm into the trunk. Each sensor was vertically staggered and located 15 cm from each other on the north side of the tree. A dimensionless flow index (*K*), was calculated from the difference in temperature (*T*) measured between the two probes following Granier (1987) and can be expressed as:

1 $$ K=\frac{\Delta T\max -\Delta T}{\Delta T} $$

*K* values were calculated by determining zero-flow conditions using the open-source software Baseliner (Oishi et al. 2016). Measurements were collected continuously from 14 August to 20 August 2019 and averaged into half-hour intervals.

Sap flux density (mL m^−2^_sapwood_ s^−1^) was calculated following Granier (1987) and using the original coefficients, as a species-specific calibration was not conducted. *J*_*s*_ was then scaled up to whole-tree water use (L d^−1^) by multiplying by the cross-sectional sapwood area (*A*_*s*_).

To estimate error, whole-tree water use was calculated both accounting for radial variation (*Q*_*r*_) and assuming uniform *J*_*s*_ (*Q*_*u*_). When calculating *Q*_*u*_, only the sap flux density measurements made in the outermost depth interval (0–20 mm) were used to scale up to the whole-tree level.

### Determining sapwood depth

Sapwood depth, heartwood depth, and total xylem depth (from the pith to the xylem edge) were measured using a 5.15-mm increment borer and identified visually based on coloration changes between sapwood and heartwood. A total of 34 cores were taken in 17 trees. Each tree was cored twice to obtain average depth per tree. Cores were collected from trees not instrumented with sap flow sensors to prevent disruption to the hydraulic conductivity of the sapwood.

### Integration of non-uniform sap flux density

*J*_*s*_ (mL m^−2^_sapwood_ s^−1^) was averaged at each depth interval between the three sample trees and a daily average was computed over the seven-day study period. We then calculated the area under each daily *J*_*s*_ curve to get daily water flux (cm of water) at each of the three depth intervals. Once the average daily water flux was calculated at each depth within the sapwood, a fourth-order polynomial trend line was fit to the data. The following assumptions were made: (i) no hydraulic flow within the heartwood and (ii) flow at the edge of the cambium (not measured) is 70% of the measured velocity in the outermost depth interval. Assumption ii was based on similar findings from several *Pinus* species reported in Nadezhdina et al. (2002) and Ford et al. (2004b)*.* Finally, the polynomial was integrated between 7 and 14 cm to represent the average daily volume transpired (cm^3^).

### Study limitations

Limitations of our study include a small sample size (*n* = 3) and temporally short duration of measurements. As mentioned earlier, this small subset of sensors reported in this article is part of a larger ongoing experimental study comparing transpiration between various forest management techniques. Due to uncertainties associated with the potential disruption of hydraulic flow in trees with multiple sensors, the sample size of this radial study was kept limited so as not to compromise the integrity of the larger study. The short duration of measurements was caused by disruption to the power supply for all sensors, lasting more than 1 month.

## Results

### Meteorological conditions

**Fig. 1:**
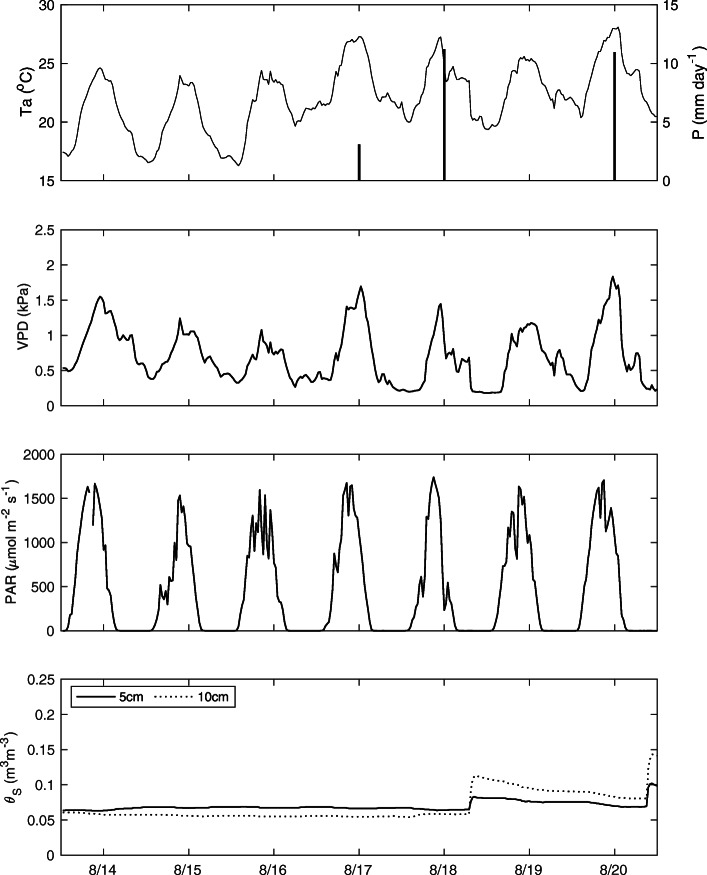
Meteorological measurements of **a** air temperature (Ta) and precipitation (P), **b** vapor pressure deficit (VPD), **c** photosynthetically active radiation (PAR), and **d** soil water content (θ) from August 14 to 20, 2019

The study period experienced average daily temperatures when compared to the 30-year mean climate record for this region. Precipitation occurred on August 17, 18, and 20 (Fig. 1a) with a total value of 25 mm during the study period. *J*_*s*_ followed a similar diurnal pattern as air temperature and vapor pressure deficit (VPD; Fig. 1b), suggesting transpiration at this site is driven primarily by temperature and VPD.

### Relationship between sapwood area and diameter

Previous studies have developed species-specific allometric equations relating sapwood area to tree diameter (Bovard et al. 2005; Matheny et al. 2014; Skubel et al. 2017), but no such relationship is known for red pine or *Pinus resinosa*. This study developed an allometric equation (Eq. 2) relating sapwood area (*A*_*s*_) to tree diameter at breast height (DBH).

2 $$ {\mathrm{A}}_{\mathrm{s}}={\mathrm{aDBH}}^{\mathrm{b}} $$

**Fig. 2:**
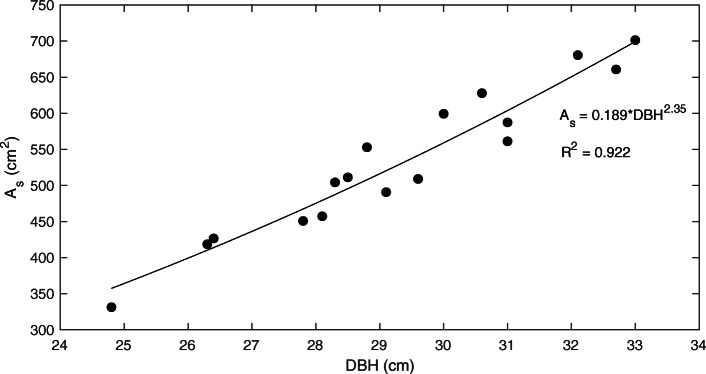
A species-specific allometric equation relating sapwood area (*A*_*s*_) to tree diameter measured at breast or 1.3 m height (DBH)

The species-specific parameters (*a*, *b*) of the allometric equation are displayed in Fig. 2 and report an *R*^2^ value of 0.92.

### Radial profiles of sap flux density

**Fig. 3:**
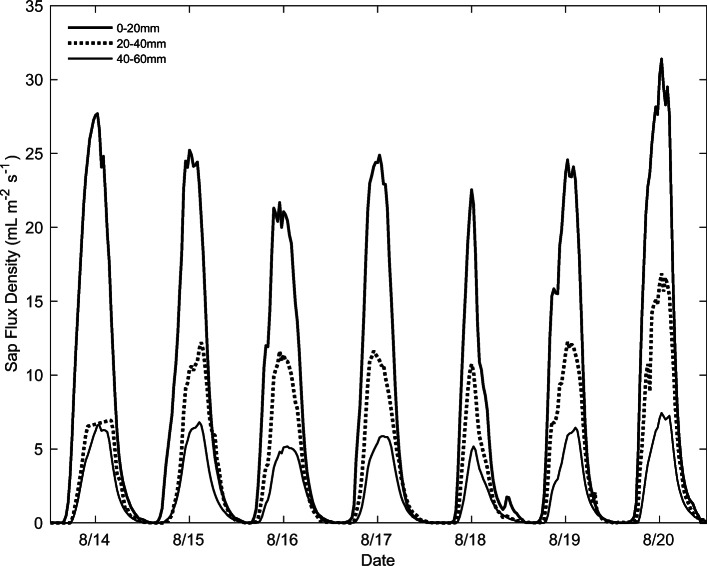
Diurnal patterns of sap flux densities (*J*_*s*_) at different depth intervals within the sapwood from August 14 to 20, 2019

As expected, sap flux density was greatest in the outermost 0–20 mm of sapwood and decreased toward the pith. Figure 3 illustrates the average of all sensors at each depth for the duration of the study period. All sensors exhibited the same diurnal pattern suggesting both a level of accuracy and similar timing of flow, regardless of depth. *J*_*s*_ peaked between 12:00 and 14:00 each day of the study period. The difference in *J*_*s*_ between each of the depth intervals was consistent, except for August 14 in which *J*_*s*_ in the middle (20–40 mm) depth was not significantly different to the innermost (40–60 mm) depth. This could be due to a brief power issue, as it was seen among all sensors.

The average daytime (between 8:00 and 20:00) *J*_*s*_ was 14.8, 7.1, and 3.7 mL m^−2^ s^−1^ for the outer, middle, and inner portions of the sapwood, respectively. The maximum *J*_*s*_ in the outer depth was 31.4 mL m^−2^ s^−1^ on 20 August (13:00), while the innermost depth reached a maximum of 7.4 mL m^−2^ s^−1^ on the same day/time.

**Fig. 4:**
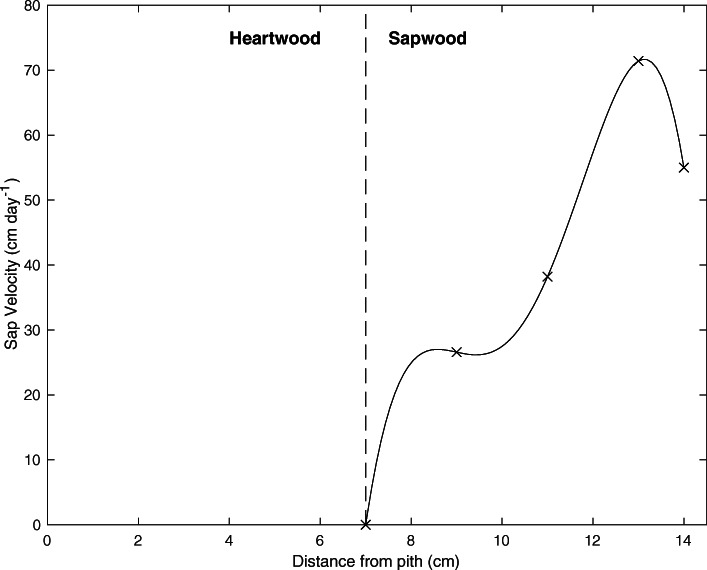
Daily mean sap velocity at varying depth from the pith. On average, the heartwood extends approximately 7 cm from the pith. At 7 cm, the sapwood begins and extends to 14 cm where it reaches the edge of the cambium

Figure 4 shows an average daily profile of water flux throughout the conductive tissue of the sapwood, showing the measurements at 9, 11, and 13 cm from the pith and assumptions made for both the sapwood-heartwood border and the edge of the cambium. The relationship is best described by a fourth-order polynomial and indicates the highest flow at approximately 1 cm from the edge of the cambium.

### Errors associated with assuming uniform sap flux density

On average, sap flux at the 20 to 40 mm, and 40 to 60 mm depths accounted for approximately 56 and 32% of that at the 0–20 mm depth, respectively. These results indicate that most of the water transport occurred in the outermost sapwood.

By failing to account for radial variation in *J*_*s*_, the results show that whole-tree water use is overestimated by as much as 81% (Table 2). When possible, it is therefore favorable to measure *J*_*s*_ at various points in the conductive sapwood to report whole-tree transpiration.

**Table 2:** Estimates of error in calculating daily whole-tree water use when assuming uniform sap flux density

Sample no.	*Q*_*r*_ (L)	*Q*_*u*_ (L)	Error ($\frac{Qu- Qr}{Qr}$**) (%)**	Mean error (%)
1	13.09	20.63	57.6	61.4
2	3.02	5.47	81.1	
3	8.79	12.78	45.4	

## Discussion

The measurement of sap flow is a widely adopted technique to quantify transpiration and water use in vegetation ecosystems including forests. Sap flux density substantially varies within the sapwood of trees and among different forest species. Therefore, to better quantify ecosystem water fluxes, variations in hydraulic conductivity at the tree-level must be considered in forest ecosystems. Our study is one of the first to explore radial sap flux variability in the red pine (*Pinus resinosa*) forests. We found that most of the sap flux occurred in the outermost sapwood, within 3 cm from the cambium, validating our proposed hypothesis. In the literature, Delzon et al. (2004) had explored a similar hypothesis in other species of pine and found that by neglecting the radial variation within the sapwood, transpiration was overestimated by up to 47%. Other studies have suggested that this overestimation can be as large as 300% (Nadezhdina et al. 2002; Ford et al. 2004b). Although for many species sap flux density is highest near the cambium, Čermák et al. (1992) found a Gaussian distribution of sap flux density with depth in Norway spruce (*Picea abies*). Radial variation is, therefore, dependent on wood- and species-type (Berdanier et al. 2016) and is widely agreed that this variability needs to be addressed when scaling sap flow measurements to the tree- and stand-level.

### Variability in radial depth profiles

Radial depth profiles have been shown to vary diurnally (Ford et al. 2004b; Poyatos et al. 2007), and with changes in soil water content (Ford et al. 2004a). The study period in which our research was conducted did not show significant changes in soil water content (*θ*; Fig. 1d) to investigate the latter. In fact, studies have shown that depth profiles remained relatively constant during periods of stable soil moisture conditions (Lu et al. 2000). Variability between trees and within a single tree has also been studied to determine the need to establish a depth profile for individual trees (Lu et al. 2000; Delzon et al. 2004; Kumagai et al. 2005). In our study, we do not examine differences in depth profile within an individual tree due to the highly symmetrical distribution of sapwood cross-sectionally in this plantation forest.

### Relationship between basal diameter and sapwood area

Our study developed an allometric relationship between tree diameter (DBH) and sapwood area for our red pine stand as shown by Eq. 2. Other studies have similarly established allometric equations for several other species (Matheny et al. 2014; Bovard et al. 2005) but our study is one of the first for red pine forests. This relationship, which is highly species-specific, can be used in future studies when determining the positioning of sap flow sensors and scaling point-measurements to the tree- and stand-levels.

### Quantification of errors when scaling to tree level

Studies in the literature have shown differences in radial depth profile depending on wood-type (Phillips et al. 1996). For instance, Čermák et al. (1992) found the radial profile in Norway spruce (*Picea abies*, non-porous) to be symmetrical, with sap velocity peaking at the midpoint of the sapwood depth. In contrast, they found an assymetrical distribution of sap flow in oak (*Quercus robur*, ring-porous) (Čermák et al. 1992). However, Phillips et al. (1996) found sap flux density to decrease from the cambium to the pith in loblolly pine (*Pinus taeda*, non-porous), which more similarly reflects the results from our study.

In Scots pine (*Pinus sylvestris*), Nadezhdina et al. (2002) showed that the majority of sap flow occurred at a depth of 85 to 95% of the xylem radius. When positioning a single sensor at this depth, sap flow was greatly overestimated by as much as 300% (Nadezhdina et al. 2002). Other studies on slash pine (*P. ellioti*), shortleaf pine (*Pinus echinata*), longleaf pine (*P. palustris*), and loblolly pine (*P. taeda*) (Ford et al. 2004a, 2004b) have given a similar distribution of sap flow. Our study has shown that the majority of water flow in red pine trees occurs in the outer depth of sapwood, closest to the cambium. This pattern of sap flow is consistent with observations made on Scots pine, slash pine, and loblolly pine as discussed above.

Previous studies in the literature have also examined the variability in conductive tissue of the eastern white pine (*P. strobus*), which is a native conifer species naturally grown or planted in the Great Lakes region. They found that the non-symmetrical nature of sapwood area increased the margin of error associated with estimating the distribution of sap flow in white pine (McIntire 2018). As compared to red pine, white pine trees had 50–70% less sapwood area (Matheny et al. 2014; McIntire 2018), which highlighted the importance of estimating the spatial variation of sapwood and hence sap flow in individual tree species.

To achieve more accurate results when scaling up sap flux density measurements, it is ideal to have multiple measurements at various depths within the sapwood of each individual tree. However, due to time, financial, and wounding constraints, in most sap flow measurement studies, such an approach is not adopted. Zang et al. (1996) and Delzon et al. (2004) investigated radial variation in sap flow and developed a correction factor adjusting a single measurement to better reflect the actual sap flux on a given day. However, research has shown there may be high temporal variations in radial depth profiles, indicating that radial depth profiles may change according to tree size (Ford et al. 2004a, 2004b), soil water availability (Lu et al. 2000; Ford et al. 2004a; Nadezhdina et al. 2007) or evaporative demand (Ford et al. 2004b). Therefore, multiple measurements within the sapwood is a preferred methodology for more accurate sap flow results as suggested by the results of our study.

In Canada, fast-growing red pine forests are an important source of softwood for the lumber industry and are widely planted. Red pine logs are straight and are extensively used in the construction industry and for electricity poles (Nature Conservancy Canada 2020). The red pine plantation forests in the Great lakes region and Eastern North America are susceptible to increasing temperatures and more frequent drought and heat events due to climate change. Therefore, the results of our study will help in the understanding of water use strategies of red pine forests. They will be useful to researchers, forest managers, and ecological policy-makers.

## Conclusions

Our study results show that transpiration from dominant red pine trees contributed significantly to the overall water balance of the forest. Overall, 65% of water transport occurred in the outer 20 mm of the sapwood, while 26% and 9% of water transport occurred in the 20–40 mm and 40–60 mm depth intervals, respectively. Furthermore, our study reveals that by failing to account for radial variability in sap flow, whole-tree water use may be over-reported by as much as 81%. These results suggest the best practices for scaling sap flow measurements to the tree- and stand-level involve measuring hydraulic flow at various depths within the conductive tissue. Overestimating stand-level transpiration can have significant implications for hydrological processes and water budgets in red pine stands in the Great Lakes region and Eastern North America. Our study will help to identify physiological traits including water storage which may predict the response of the forest to extreme events such as drought. It will also help to improve the simulation of transpiration and its upscaling from tree- to ecosystem-level and regionally.

## Data Availability

The datasets used during this study are available from the authors upon request.

## Abbreviations

*A*_*s*_: Sapwood area [cm^2^]
DBH: Diameter at breast height [cm]
*F*: Sap flow [mL s^− 1^]
*J*_*s*_: Sap flux density [mL m^−2^_sapwood_ s^− 1^]
*K*: A dimensionless flow index describing the relationship between average flow and zero flow (nighttime) conditions
PAR: Photosynthetically active radiation [μmol m^− 2^ s^− 1^]
*Q*_*r*_: Whole-tree water-use calculated by accounting for radial variation in sap flux density [L day^− 1^]
*Q*_*u*_: Whole-tree water-use calculated by assuming uniform flow throughout the sapwood [L day^− 1^]
SWCR: St. Williams Conservation Reserve
TPFS: Turkey Point Flux Station
TP31: Turkey Point pine plantation forest planted in 1931
TP39: Turkey Point pine plantation forest planted in 1939
TP74: Turkey Point pine plantation forest planted in 1974
TP02: Turkey Point pine plantation forest planted in 2002
TPD: Turkey Point mixed deciduous forest stand
VPD: Vapor pressure deficit [kPa]
VRH: Variable retention harvesting
